# Geometric Regularized Hopfield Neural Network for Medical Image Enhancement

**DOI:** 10.1155/2021/6664569

**Published:** 2021-01-22

**Authors:** Fayadh Alenezi, K. C. Santosh

**Affiliations:** ^1^Department of Electrical Engineering, College of Engineering, Jouf University Sakaka, 72388, Saudi Arabia; ^2^Department of Computer Science, University of South Dakota, Vermillion, SD 57069, USA

## Abstract

One of the major shortcomings of Hopfield neural network (HNN) is that the network may not always converge to a fixed point. HNN, predominantly, is limited to local optimization during training to achieve network stability. In this paper, the convergence problem is addressed using two approaches: (a) by sequencing the activation of a continuous modified HNN (MHNN) based on the geometric correlation of features within various image hyperplanes via pixel gradient vectors and (b) by regulating geometric pixel gradient vectors. These are achieved by regularizing proposed MHNNs under cohomology, which enables them to act as an unconventional filter for pixel spectral sequences. It shifts the focus to both local and global optimizations in order to strengthen feature correlations within each image subspace. As a result, it enhances edges, information content, contrast, and resolution. The proposed algorithm was tested on fifteen different medical images, where evaluations were made based on entropy, visual information fidelity (VIF), weighted peak signal-to-noise ratio (WPSNR), contrast, and homogeneity. Our results confirmed superiority as compared to four existing benchmark enhancement methods.

## 1. Introduction

Artificial intelligence is one of the most celebrated fields in science. Key components in artificial intelligence are neural networks, which have been integrated into image processing and computer vision. Integrating neural networks and other mathematical computation tools into computer science can be useful because they enable the exploitation of a wide range of applications related to image classification, driving automation, and text translation [1]. In this paper, a proposal aimed at integrating the benefits derived from modifying the Hopfield neural network (HNN) with geometric algebra to improve image quality is presented and evaluated.

HNNs, just like other neural networks, have contributed to the growth of various image processing tasks. They have been used widely in the detection of image features such as in quantification and segmentation [2], in feature tracking of satellite images [3], and in a variety of image restoration techniques [4]. However, HNNs have had limited success in image enhancement and have displayed several shortcomings compared to other techniques [5]. One of the common problems with HNN-based image enhancement is that it converges to a fixed point, which makes it focus on local optimization during the training stage in order to ensure the stability of the network [6]. This improves optimization processing. It, however, could hinder the quality of the processed image [7].

In order to achieve a highly reliable performance, many training data sets are also required; however, this may limit its application in some areas [8, 9]. The premise of the current proposal is that combining HNN with geometric properties of input images may expand the usability of HNN-based imaging [10]. Previous results have shown that this improvement is still an ongoing process [11–17]. One solution is to integrate the geometric hyperplane complex properties of an image with a modified HNN to optimize the mapping of image features such as edges and other physical properties. This will help improve image quality in terms of human visual perception.

Human visual perception defines image quality based on image features [18]. Image quality depends on the positioning of pixel arrays within the image dimensional space, where each dimension corresponds to a specific feature [19, 20]. Images with a higher number of pixels within the dimensional space pose problems during enhancement, leading to either poorly formed features or reduced information content [21]. The creation of a high-quality image based on human visual perception requires the alignment of correlated pixels within and among adjacent local and global neighborhoods [22]. Such alignment is based on the global and local adaptation of the reference image axis during image enhancement [23]. Therefore, a suitable computation results in better edge preservation, low errors, high signal-to-noise ratios, and a high conformance with the original image. The majority of image enhancement techniques are based on either global or local image features. These techniques result in high errors [11], a low conformance with the original image [12], or lower information than the original image [15]. Histogram-based image enhancement methods are the most commonly used, while those based on artificial neural networks (ANNs) are rarely applied. ANN-based image enhancement techniques are inspired by the biological neural networks that constitute the human brain [13]. They provide better outcomes than most other computational methods. Image enhancement techniques based on ANNs have been affected by optimization issues, which lead to noisy images with poorly formed edges, as well as a low similarity and information content in reference to the original image. Geometric-based image enhancement techniques, on the other hand, such as that of [24], focus on improving edges and structural similarities between the original and final output. [25] uses geometric mean filtering to reduce Gaussian noise present in wireless capsule endoscopy (WCE) images. The results in [25] were only compared with those achieved via arithmetic mean filtering, and the outcomes show improved qualities in terms of signal-to-noise ratio (SNR) and peak signal-to-noise ratio (PSNR). [26] used geometric parameters on local pixel intensity distributions in a novel anisotropic diffusion method for radiography image enhancement. [26] combined noise reduction, edge preservation, and sharpening operations, and the authors compared the results with only those from other anisotropic diffusion methods. In summary, the existing image enhancement methods lack proven human visual perception features since none of the current approaches focus on improving all image qualities [27]. Multiple scholars have attempted to solve these problems by introducing preprocessing [28–30] and regularizing filters [31].

Recent studies based on regularized or modified HNNs have been used extensively in image restoration, especially in confocal microscopy [32]. Modifying an HNN with other mathematical concepts, like geometric algebra, allows the network to concentrate on contextualizing information about pixels in a neighborhood [33]. Geometric algebra, particularly cohomology, allows for the exploitation of the image hyperplane; hence, it yields information-rich output images with clear edges. Therefore, the idea of regularizing HNNs under cohomology concepts technically enables HNNs to act as unconventional filters for image pixel spectral sequences [34]. The existing research has attempted to solve the fixed-point convergence problem of HNN. For instance, Tsang et al. [35] investigated and proposed updating rules associated with the convergence theorems associated with a discrete Hopfield neural network (DHNN) with delay. The serial and parallel mode updating rule proposed resulted in a faster speed than any existing rules. Hillar et al. [36] used minimum probability flow (MFP) on discretized Hopfield neural network for grayscale digital photography. The MPF surpasses the convergence problem associated with HNN, thus allowing high-quality regime compression of digital images. Kasihmuddin et al. [37] attempt to solve the minimum energy problem associated with confinement to limited solution spaces of neurons by combining the estimation distribution algorithms' global search capacity. The combination resulted in HNN exploring other solution spaces, which led to the estimation of possible neuron states to yield minimum global energy. Nour-eddine et al. [38] solved the fluctuation behaviors resulting from hard limit activators by setting parameters to settle a stable network. The current study proposes this new piece of knowledge by demonstrating its significance on image enhancement through practical examples. HNNs are preferred to traditional artificial intelligence due to their ability to allow for structural modifications and extensions for feature enhancement and pattern emphasis. Therefore, HNNs remove chances of disordered geometrical formations. HNNs' ability to retrieve and recognize features and patterns lies in the cost function, which operates similar to that of a Hamiltonian function (the minima of a Hamiltonian match similar patterns). This makes HNNs robust since the cost function can be modeled to fit various applications, such as the recognition and separation of pixel correlations within neighborhoods in order to enhance image quality [39]. This is made possible by minimizing the cost (energy) function. The mechanical extension of relativity permits HNNs to alienate free energy (loose or uncorrelated pixels) from closed or correlated pixels in the image. Altering the sign of the HNN cost function by mechanical analogy makes Hopfield's piecewise function appealing for research in other areas and has significantly influenced this paper.

The operation of a classical HNN is based on a simple quadratic energy function. Its periodic update via dynamic parameters iteratively minimizes the energy until it converges to a minimum, which corresponds to the geometric correlation of image pixels. Many different learning rules can be used to attain this outcome; however, a traditional HNN lacks the means for modeling real-world, higher-order dependencies [40] such as pixel correlation, and hence, the energy function needs to be modified. For instance, images (whether gray or red, green, blue (RGB)) have large dimensions, which makes simultaneously modeling global and local geometric dependencies difficult. Many existing HNNs can model perceptual data efficiently without interfering with model fidelity by using either existing algorithms or machine learning tools [39].

The rest of the paper is organized as follows.  Section 2 summarizes the contribution of the paper.  Section 3 outlines the background information of the Hopfield neural network and geometric algebra, specifically cohomology and residues, and then summarizes the proposed geometric regularized Hopfield neural network for image enhancement.  Section 4 describes the detailed experimental procedures, results, and comparison of the proposed results with the existing state-of-the-art results. Finally, Section 5 presents the theoretical and experimental conclusion.

## 2. Contribution Outline

In this paper, the need for a more efficient protocol for processing high-dimensional geometric dependencies in order to allow for global and local pixel correlations is recognized and addressed. The paper presents a modification of the HNN based on the geometric correlation of pixels with the goal of improving the pixel gradient vector. This optimizes the local energy function and improves the image information content while preserving image features.

## 3. Materials and Method

### 3.1. Materials

#### 3.1.1. Hopfield Neural Networks

**Figure 1:**
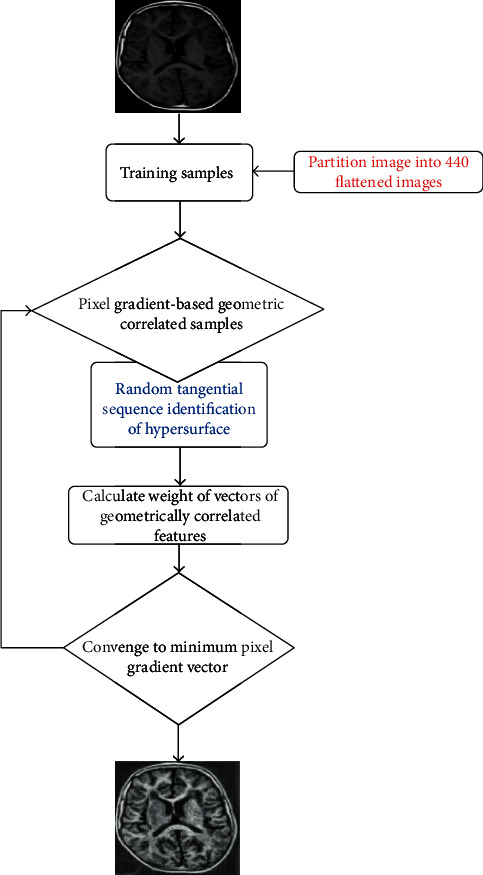
Schematic diagram indicating key steps of the proposed algorithm for the results presented in Figures 3 and 4. The MHNN proposed is composed of 4 layers: one input layer, two hidden layers, and one output layer.

In this paper, a new method for image enhancement is presented (see Figure 1). HNNs have two significant limitations: the learning process and the convergence process [41]. These limitations often lead to the alteration of geometric correlations. The HNN classification process depends on an energy function and therefore aims at reaching local rather than global geometric minima [42]. This tendency creates correlation problems, especially when single images are used as inputs, as is the case in the algorithm proposed in this paper. This limitation has surpassed the primary efforts based on HNNs alone [43] in the areas of image restoration, segmentation, and object classification. Various modifications of HNNs by different researchers have also shown significant limitations in terms of the extraction of the learning vector space and, therefore, have often led to the wrong choice of vector space [4, 44]. Modified Hopfield neural networks (MHNNs) are also time consuming due to the presence of looping and self-connecting architectures. Neither MHNNs nor HNNs have been validated, and both yield noisy results, which make them unsuitable in terms of improving image perceptual quality [41].

In this paper, a novel method based on a geometric MHNN aimed at improving the human perceptual quality of images is presented and evaluated. Unlike existing methods, this method focuses on addressing the disadvantages associated with existing MHNNs, as well as basic and continuous HNNs in order to improve image quality. The proposed MHNN, unlike any other method previously proposed, considers a geometric correction of pixels within an image neighborhood, so that the usual focus of HNNs (minimizing energy) is replaced by the search of a global optimum to help improve image quality.

**Figure 2:**
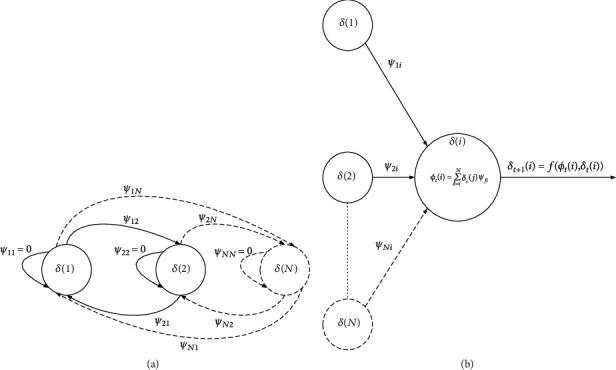
(a) HNN and (b) propagation rule and activation function.

HNNs are iterative, autoassociative networks that consist of a single layer of processing elements, so they are categorized as associative memory [45]. HNNs are categorized into recurrent and fully connected neural networks and have two versions: binary and continuous [45]. In the binary version, all of the neurons are connected to each other, but there is no self-connection. The continuous version allows for all possible connections [45]. The *N*-node HNN parlance is an *N*-dimensional vector *ξ* = [*ξ*_1_, ⋯, *ξ*_*N*_] from the space *Ξ* = {−1, 1}^*N*^. A special subset of *Ξ* represents the reference pattern Γ = {*γ*^*η*^ : 1 ≤ *η*≤∞}, where *γ*^*η*^ = [*γ*_1_^*η*^, ⋯, *γ*_*N*_^*η*^]. HNNs link a vector from *Ξ* into classes whose members have similar characteristics to the reference subset Γ. Just like any other neural network, HNNs have the following basic components:

1. A finite set of neurons *δ*(*i*), 1 ≤ *i* ≤ *N*, which serve as processing units and are described by the value or state *δ*_*t*_(*i*) at time *t*. The state can either be −1 or +1 and are therefore represented as *δ*_*t*_(*i*) ∈ −1, +1 [45, 46]
2. A synaptic connection where the learned information of the neural network resides, which is defined as interconnections between neurons. A synaptic connection *ψ*_*ij*_, which exists between any two neurons *δ*(*i*) and *δ*(*j*) such that *ψ*_*ij*_ = *ψ*_*ji*_ and *ψ*_*ij*_ = 0 for *j* = *i*, as portrayed in Figure 2(a) [45, 46]. Synaptic changes in the network for the case of continuous HNNs are nonexistent, and excitation and inhibition is achieved by means of a weighted sum of the contributions of the neighboring neuron outputs
3. A propagation rule, as presented in Figure 2(b), defines how states and synapses influence the input of each neuron [46] as follows:

(1) $$\begin{array}{c} \phi_{t}\left(i\right)=\overset{n}{\underset{j=1}{\sum }}\delta_{t}\left(j\right)\psi_{ij}+{℘}_{i}, \end{array}$$

where ℘_*i*_ is the neuron bias, which depends on external conditions

(4) An activation function *f*, which determines the subsequent state of neuron *δ*_*t*+1_(*i*) based on the propagation value *ϕ*_*t*_(*i*) computed using (1) and the current state of neuron *δ*_*t*_(*i*). The activation function is accomplished by the network as it attempts to learn patterns that are *N*-dimensional vectors from image space *Ξ*, where *Ξ* ∈ [−1, 1]^*N*^. Defining *γ*^*η*^ = [*γ*_1_^*η*^, *γ*_2_^*η*^, ⋯, *γ*_*n*_^*η*^] as the *ξ*^th^ exemplar pattern, where 1 ≤ *η* ≤ *ζ*. Then, the dimensionality pattern space of the HNN is reflected in the number of nodes in the network in Figure 2(b) and in the network with *N* nodes *δ*(1), *δ*(2), ⋯, *δ*(*N*) [45, 46]:

(2) $$\begin{array}{c} \delta_{t+1}\left(i\right)=f\left(\phi_{t}\left(i\right),\delta_{t}\left(i\right)\right)=\left(\begin{array}{cc} 1, & \phi_{t}\left(i\right)>0,\\ -1, & \phi_{t}\left(i\right)<0 \end{array}\right. \end{array}$$

The basic HNN training process entails four steps:

1. Learning: this step involves assigning weights *ψ*_*ij*_ to all synaptic connections:

(3) $$\begin{array}{c} \psi_{ij}=\left(\begin{array}{cc} \overset{\zeta }{\underset{\eta =1}{\sum }}\gamma_{i}^{\eta }\gamma_{j}^{\eta }, & i\neq j\\ 0, & i=j. \end{array}\right. \end{array}$$

Keeping in mind that *ψ*_*ij*_ = *ψ*_*ji*_, that is, weights are symmetric, the preceding computation needs only be performed for *i* < *j*

(2) Initialization: this is where the pattern is presented to the network based on the similarity from the learning process such that if *ξ* = [*ξ*_1_, *ξ*_2_, ⋯, *ξ*_*N*_] is the unknown patterns, then set the initial state defined by [45, 46] is

(4) $$\begin{array}{c} \delta_{0}\left(i\right)=\xi_{i},1\leq i\leq N \end{array}$$

(3) Adaptation: this is iterative learning convergence where (1) and (2) are used to obtain the next state defined by [45, 46]:

(5) $$\begin{array}{c} \delta_{t+1}\left(i\right)=f\left(\overset{N}{\underset{j=1}{\sum }}\delta_{t}\left(j\right)\psi_{ij},\delta_{t}\left(i\right)\right) \end{array}$$

(4) Continuation: this step represents the repetition of steps 2 and 3. The iterative learning continues until no further changes are observed in the state of any node

The steps outlined above are common and remain similar for all HNNs. However, there are some variations in the continuous version such as in image processing (where *ψ*_*ij*_ ≠ 0) and in the case of the sigmoid function in the activation function, as defined by [45, 46]:

(6) $$\begin{array}{c} f\left(\delta \left(i\right)\right)=\frac{1}{1+e^{-\delta \left(\phi_{i}-\iota \right)}}, \end{array}$$

where *ι* controls the shift along the horizontal axis. The convergence property of the basic HNN depends on the structure of Ψ (the matrix with elements *ψ*_*ij*_) and the update model. One of the main advantages of the basic HNN is the operation in sequential mode, where Ψ is symmetrical with nonnegative diagonal elements. Thus, the energy function is defined by [45, 46]:

(7) $$\begin{array}{c} E\left(t\right)=\frac{1}{2}\overset{n}{\underset{i=1}{\sum }}\overset{n}{\underset{j=1}{\sum }}\psi_{ij}\delta_{j}\left(t\right)\delta_{i}\left(t\right)-\overset{n}{\underset{i=1}{\sum }}{℘}_{i}\delta_{i}\left(t\right). \end{array}$$

*E*(*t*) in (7) is a Lyapunov function. It is nonincreasing and converges to a fixed point. The energy function in (7) represents the overall status of the network [46]. Energy values increase at each iteration and become stable when (7) reaches its minimum [40].

#### 3.1.2. Geometric Algebra: Cohomology and Residues

**Figure 3:**
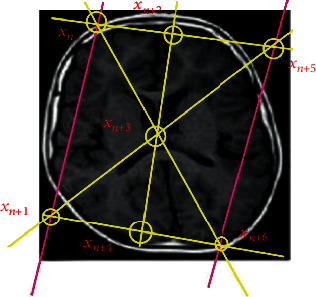
A visual of a geometric Lefshetz hyperplane in an image.

The difference between continuous and chain maps in the bijection between the categories in the image geometric hyperplane represented in Figure 3 can be summarized as follows;

- Continuous map is often represented by {**C**_**n**_(**X**), *∂*_**n**_} while chain is *𝒞* = {**C**_**n**_, *∂*_**n**_}
- Continuous maps function shows **f** : **X** → **Y** while chain maps functions summarizes **f** : *𝒞* → *𝒟*

where {**C**_**n**_(**X**), *∂*_**n**_} represent image pixels, *x*_*i*_, *y*_*i*_ and {**C**_**n**_, *∂*_**n**_}≃*x*_*n*_, *y*_*n*_. This can be simply interpreted as a geometric correlation existing between pixels within an image subspace. This suggests that geometric mapping of these points via the Lefshetz formula links local and global features of the image. The homotopy and chain homotopy suggests that

(8) $$\begin{array}{c} A.A=detA.\text{I}=\left[\begin{array}{cccc} detA & & 0 & \\ & detA & & \\ & & \ddots & \\ & 0 & & detA \end{array}\right]. \end{array}$$

We use this odd formalism to understand how to compute the Euler characteristic (the middle cohomology) of a smooth image projective hypersurface in *ℙ*^*n*+1^. The first step is to identify the *tangent sequence for a hypersurface* [47] of an image to ensure geometric correlations are enhanced, that is,

(9) $$\begin{array}{c} 0\to T_{X}\to T_{{\left.{\mathbb{P}}^{n+1}\right|}_{X}}\to O_{X}\left(d\right)\to 0. \end{array}$$

It is possible to go one step further to give a more explicit description of the cohomology of a smooth image hypersurface **X**_*d*_ ⊂ *ℙ*^*n*+1^. To do this, consider **H**^*n*^(**X**, *ℤ*) and its complexity **H**^*n*^(**X**, *ℂ*). By the Lefschetz theorem on a hyperplane, decomposition of **H**^*n*^(**X**, *ℂ*)≅**H**^*n*^(*ℙ*^*n*+1^, *ℂ*) ⊕ **A** is plausible. The summed **A** is called the primitive cohomology of **X** and is denoted by **H**_**prim**_^**n**^(**X**). Next, a question arises of whether there is any relation between this primitive cohomology and the ambient space *ℙ*^*n*+1^ of an image to ensure a sequence of closed subvarieties of image global and local features exist. The answer is determined based on the following theorem [48].

**Theorem 1 .**

Let **X**_*d*_ ⊂ *ℙ*^*n*+1^ be a smooth hypersurface of degree *d*. The **H**_**prim**_^**n**^(**X**, *ℂ*) is generated by [47]

(10) $$\begin{array}{c} {\left\{\text{Res}\left(\frac{Adx_{0}dx_{n+1}}{f^{p}}\right)\right\}}_{p=0}^{n}, \end{array}$$

where

(11) $$\begin{array}{c} \text{Res}\left(\frac{Adx_{0}dx_{n+1}}{f^{p}}\right)=2\pi i\sum A\frac{{\left(dx_{0}dx_{n+1}\right)}^{i_{1}i_{p}}}{f_{i_{1}}f_{i_{p}}} \end{array}$$

and *A* ∈ *R*_*pd*−*n*−2_.

More precisely, one can find that

(12) $$\begin{array}{c} H_{\text{prim}}^{n}\left(X,\mathbb{C}\right)≅\overset{n}{\underset{p=0}{⊕}}{\left(M_{F}\right)}_{\left(p+1\right)d-n-2}. \end{array}$$

Finally, a simple but enlightening application of these results in relation to the proposed algorithm can be formulated as

**Lemma 1 .**

Let *Q* be a smooth quadratic hypersurface in *ℙ*^*n*+1^. Then,

(13) $$\begin{array}{c} X\left(Q\right)=\left\{\begin{array}{cc} n+3, & n+1\text{ is odd},\\ n+2, & n+1\text{ is even}. \end{array}\right. \end{array}$$

**Proof**

Use a matrix of signs to determine (−1)^*i*+*j*^ as the sign sequencing activation function in proposed HNN.

(14) $$\begin{array}{c} \left[\begin{array}{cccc} + & - & + & \\ - & + & - & \\ + & - & + & \\ \vdots & \vdots & \vdots & \ddots \end{array}\right]. \end{array}$$

Therefore, we have ker(*∂*_1_) = 〈*x* + *y* − *z*〉≅*ℤ*.

### 3.2. Geometric Regularized Hopfield Neural Network for Image Enhancement

Improving human visual perception in images through the explicit operation of arrays of strongly correlated pixels is subtle and remains unaddressed. However, several studies have reported the existence of weaker constraints that can be modified in order to enhance image perception [49, 50]. The standard image quality enhancement techniques have not addressed the challenges associated with intricate features arising from failure by other methods to recognize geometric variations in pixel correlation within regions in images. Even the use of biologically inspired neural networks has not addressed the inability to match the undifferentiated range of pixel intensities and correlation within images. As a result, many techniques focus on image segmentation [44, 51], classification [52, 53], and contrast or resolution enhancement [43, 54], but none has attempted to implicitly improve image perception quality based on geometric variations in pixel correlations.

In this paper, image pixel neighborhood geometric correlations are demonstrated given that regions with similar features have high pixel geometric correlations. These pixels become highly geometrically correlated when small changes are introduced into their arrangements, and they neither obey homotopy nor chain homotopy when features become uncorrelated [55]. The details of the proposed algorithm are summarized in Figure 1 and described below.

The proposed MHNN presented in Figure 1 exploits the fact that features within an image utilize similar pixel geometric correlations [56, 57]. Such geometric correlations, therefore, can be used to model and reproduce an enhanced image with better feature representation. Finding an optimal pixel geometric correlation within image neighborhoods ensures that the final image has a better human perception. Assuming that an image region or patch consisting of *M* × *M* pixels is perceived to have some geometric correlation, image pixels can be extracted and transformed into row vectors. Consequently, the image is comprised of *N* × *N* patches, each described by a group of pixels *Λ* and each pixel *δ*^*i*^. Letting *δ*^*i*^ such that 1 ≤ *i* ≤ *N*^2^ be an *M*-dimensional feature vector for pixels within each image hyperplane, and assuming that *Λ* is a known feature, then all *δ*^*i*^ belong to *Λ*. Given a known number of hyperplanes *Λ*, discrete geometric feature pixel *δ*^*i*^, and that the position of the pixel when the maximum geometric correlation will only be achieved if (14) is true, then a feature optimization problem can be expressed as

(15) $$\begin{array}{c} \Pi \left(\delta \right)=\overset{N^{2}}{\underset{i=1}{\sum }}\Pi \left(\delta^{i},I_{1},\cdots ,I_{Q},\delta_{i}\right)-\kappa f\left(\delta \right);\text{subject to }\kappa ,\text{are gulating parameter given by}, \end{array}$$

where *δ* = *δ*_1_, ⋯, *δ*_*N*^2^_ is a set of pixels with *Q*-dimensional vectors that have a geometric correlation as represented in Figure 3 that describe features in an image hyperplane in class of vectors *δ*^*i*^. The conditions on the vector pixels *δ*_*iq*_ ∈ [0, 1] and ∑_*q*=1_^*Q*^*δ*_*iq*_ = 1∀ 1 ≤ *i* ≤ *N*^2^ are imposed. The optimal pixel gradient, *ℑ*_*q*_ : *q* = 1, ⋯, *Q*, depends on the local orientation $\hat{\chi }$. The pixels within the region are represented by *f*(*δ*) = *f*(*δ*_1_, ⋯, *δ*_*N*^2^_), and *κ* is a parameter regulating the geometric pixel gradient vectors represented by

(16) $$\begin{array}{c} {\left(\nabla \kappa^{T}\bar{\chi }\right)}^{2}={\left|\nabla \kappa \right|}^{2}cos^{2}\left(∠\left(\nabla \kappa ,\hat{\chi }\right)\right.. \end{array}$$

Equation (16) is used to emphasize the geometric orientation of the pixel gradient vector within the image neighborhood, such that

(17) $$\begin{array}{c} \int \epsilon \left(\delta -\delta^{\prime }\right){\left(\nabla \kappa {\left(\delta^{\prime }\right)}^{T}\overset{\wedge }{\chi }\right)}^{2}d^{M}\delta^{\prime }=\overset{¯}{{\left(\nabla \kappa \overset{\wedge }{\chi }\right)}^{2}}, \end{array}$$

where *ϵ* determines the size and shape of the hyperplane around *δ* (as shown in (3)). The maximum sequencing must be achieved for each *δ* within each *M*-directional local neighborhood. This will ensure that the resultant pixel has the optimum pixel geometric orientation to allow for the reconstruction of a better image. However, in order to effectively enhance images through minimum global variance and maximum local variance, the quantity in (17) is minimized within unit pixels' orientation vector $\hat{\upsilon }$ at time *t* so that (18) is minimized,

(18) $$\begin{array}{c} \int \epsilon \left(\delta -\delta^{\prime },\delta_{j}-\delta_{j}^{\prime }\right){\left(\nabla_{\delta t}\kappa {\left(\delta^{\prime }{\delta_{j}}^{\prime }\right)}^{T}\overset{\wedge }{\upsilon }\right)}^{2}d^{M}\delta^{\prime }\delta^{\prime }d\delta_{j}^{\prime }=\overset{¯}{{\left(\nabla_{\delta t}\kappa^{T}\overset{\wedge }{\upsilon }\right)}^{2}}, \end{array}$$

where *ϵ* extends to determine the size and shape of the neighborhood around the hyperplane [*δ*_*i*_, *δ*_*j*_]^*T*^ with similar pixel coordinates. Minimization of *Π* in *Π*(*δ*) based on (18) represents the optimal pixel of the image patches that has maximum perceptual quality and is feasible only if

(19) $$\begin{array}{c} \Pi \left(\delta \right)=\overset{Q}{\underset{q=1}{\sum }}{\left\|\xi_{i}-I_{q}\right\|}_{2}^{2}\delta_{iq}+\kappa \underset{j\in \mu_{i}}{\sum }\delta_{j}^{T}\delta_{i}, \end{array}$$

where *μ*_*i*_ is the set of all pixels within the image regions that are neighbors of *i* selected for enhancement.

During pixel selection, the number of neurons in the proposed HNN is the same as the number of pixels in the selected hyperplane. The energy of this proposed HNN is thus defined as

(20) $$\begin{array}{c} E=-\overset{N^{2}}{\underset{i=1}{\sum }}\overset{N^{2}}{\underset{j=1}{\sum }}\psi_{ij}\delta_{i}\left(t\right)\delta_{j}\left(t\right)-\overset{i=1}{\underset{N^{2}}{\sum }}{℘}_{i}\delta_{i}\left(t\right), \end{array}$$

where *ψ*_*ij*_ are the net weights, *δ*_*i*_ is the state of the *i*^th^ neuron and ℘_*i*_ is the bias input to the *i*^th^ neuron. A stable HNN has decreasing energy over time and is therefore useful in solving *Q*-class pixel selection using (19) where *ψ*_*ij*_ and ℘_*i*_ are estimated using (21) and (22), respectively,

(21) $$\begin{array}{c} \psi_{ij}=2\kappa \forall j\in \mu_{i},i\neq j, \end{array}$$

(22) $$\begin{array}{c} {℘}_{q}^{i}=\frac{1}{Q}\overset{Q}{\underset{q=1}{\sum }}{\left\|\xi_{i}-I_{q}\right\|}_{2}^{2}-\frac{8\kappa }{Q}-{\left\|\xi_{i}-I_{q}\right\|}_{2}^{2}, \end{array}$$

where *Q* is defined by Lemma 2. The activation function is defined as

(23) $$\begin{array}{c} \delta_{i}=\left(\begin{array}{cc} 1, & \phi >0\\ 0, & \phi \leq 0 \end{array}\right., \end{array}$$

where *ϕ* is defined in (1).

The proposed algorithms is summarized in the following steps:

1. Given the image hyperplane, as visualized in Figure 3, geometric correlated pixels are extracted and transformed into row vectors
2. *Initialization*: calculate the gradient of these vectors using ℘_*q*_ and neuron output *δ*_*i*_. Pixel vectors must be such that *δ*_*iq*_ ∈ [0, 1], and ∑_*q*_*δ*_*iq*_ = 1∀1 ≤ *i* ≤ *N*^2^, 1 ≤ *q* ≤ *Q* and ℘_*q*_ depends on local orientation $\hat{\chi }$, which is subject to (16) and (17)
3. *Repeat*: during each iteration, *η*, for each neuron, *δ*_*i*_, compute the input of the neuron using (21), which must be sequenced by (14), and stable based on (23), and obtain a hypothesis *ν*_*t*_
4. The output gives geometric correlated pixels with global and local mapped features such that the image is smooth and has better visual perception

## 4. Experiments

### 4.1. Data Set and Implementation

**Figure 4:**
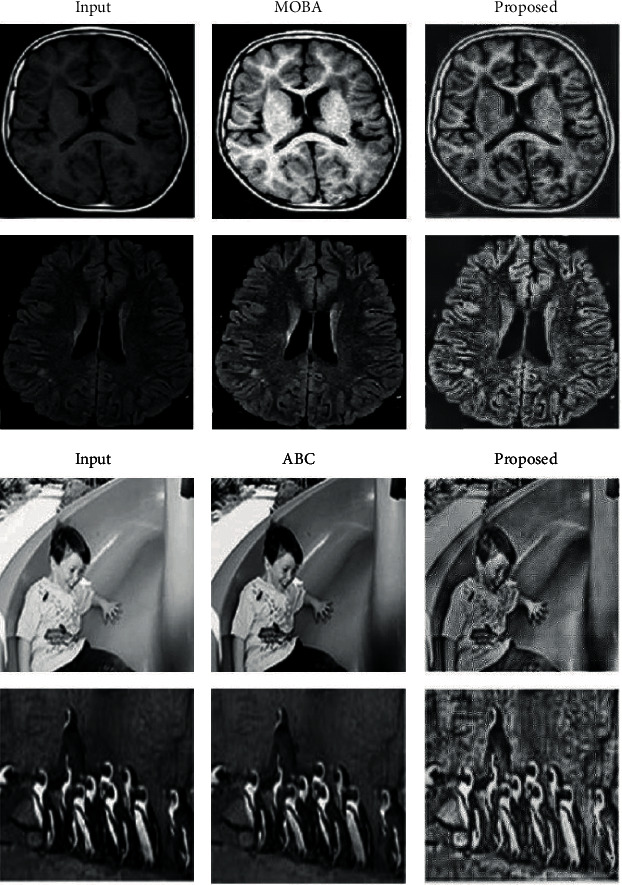
Summary of the example of the test comparison between the original image and the results from the MOBA [60]), ABC [59], and the proposed algorithm.

The proposed method uses 8-bit gray-level images with 8 layers (see Figures 3 and 4). The images were selected and proposed from the existing state-of-the-art methods [58–61]. These images were processed to 8-bit layers since HNN is a bipolar system, allowing only input data with −1 and +1.

We constructed the proposed HNN with geometric algebra defined in Section 3.1.2 where pixel patterns acted as memories. The network converged the energy function by Equation (7). The learning rule stored patterns without errors in the network. Parameters were estimated using minimum probability flow (MPF) based on the energy equation (Equation (7)), thus increasing efficiency during computation training. MPF assumed neighborhood pixels are binary vectors that are 1 unit apart, that is, 1 bit different from each other. The binary vectors of the 8-bit layer image maps (see Figure 3) are normalized based on Theorem 1. This normalization is inspired by the response property of ON/OFF of the mammalian retinal ganglion cells. The mean and variance of each pixel of the 8-bit layer image map patch were computed and normalized to 1, respectively. Each pixel intensity was then mapped onto proposed HNN based ‘ON' and ‘OFF.' Neuron firing was based on pixel intensity values, that is, lowest, middle, and highest intervals pixels inspires ‘OFF' no, and ‘ON' neuron firing, respectively. This permitted the conversion of any 8-bit gray level image into a 32-bit binary vector of abstract ‘ON' and ‘OFF' neurons. 25 image data set (with each single image having 440 partition) whose examples are presented in Figures 3 and 4 were prepared according to [62]. The training processed for the proposed HNN with *n* = 32 nodes using an optimum number of 440 partition images where MPF was used to estimate parameters on BIZON X5000 G2 with 16GB RAM was ≃8 minutes.

### 4.2. Evaluation Metrics

**Figure 5:**
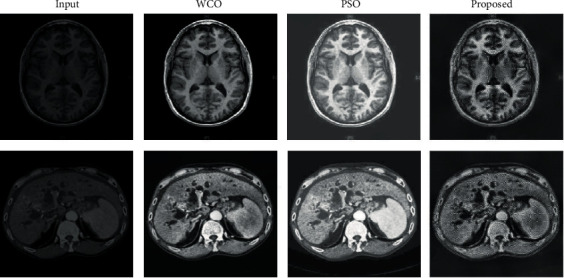
Summary of the example of the test comparison between the original image and the results from the WCO [58], PSO [61], and the proposed algorithm.

The proposed method was applied to various images (presented in Figures 4 and 5) sourced from different databases. These include MRI images of brain tumor, breast cancer, liver cancer, and skin cancer. Nonmedical images such as those of boy and penguin (see Figure 4) were also tested in order to show versatility of the proposed method. The preprocessing also included scale normalization to ensure pixel intensity values fall between 0 and 1. The final processed images, which were in partition of 440, were reconstructed to form the final image.

To evaluate our method, the following metrics were employed: entropy, visual information fidelity, weighted peak signal-to-noise ratio (WPSNR), contrast, and homogeneity. These metrics were chosen based on the objective of the proposed method, that is, improve information content, human visual quality, and textural features of an image.

1. Entropy: it is a measure of information content in an image [63]. Therefore, higher entropy is indicative of more detailed the image
2. VIF: it is similar to HVS and is based on quality assessment (QA) methods. VIF is nonnegative since it's a ratio between the original image and processed image. Therefore, higher values (i.e., VIF⟶1) are desirable and shows improvement in visual quality [64]
3. WPSNR: it is based on the human visual system (HVS) and portrays better results than peak signal-to-noise ratio (PSNR) [65]. WPSNR uses the redundancy rule of the human eye against high-frequency cases in images. Higher WPSNR values indicate higher quality of the enhanced image
4. Contrast: it is a statistical measure which results in the difference in the value between image intensity and its neighbor for the input image [58]. Higher contrast values are desirable as it indicate better visual appearance
5. Homogeneity: it is a measure of likeliness of the image intensities [58]. This measure suggests that higher values of homogeneity are desirable and indicate higher quality image

### 4.3. Result Analysis and Comparison

The proposed method was evaluated with the corresponding input image as the reference image as presented in examples in Figures 4 and 5. The state-of-the-art methods used for comparison purposes are sampled based on the similarities between the objectives of the existing works and those of the present investigation (that is, image enhancement rather than image reconstruction).  Table 1 shows the comparison performance evaluation of the proposed method in terms of mean *μ* and standard deviation *σ*. Standard deviation values shows how closely the data is to each other; hence, lower values are desirable. The tables are segmented as per the comparison method.

**Figure 6:**
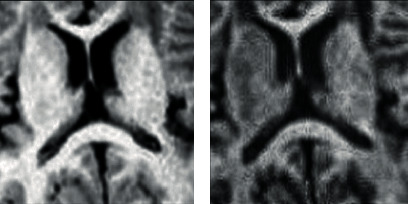
Sample of the zoomed sections comparing proposed method with existing methods.

**Figure 7:**
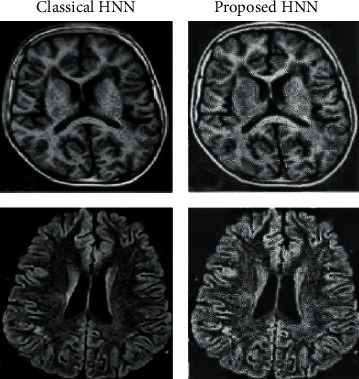
Sample comparing results produced by classical HNN with proposed.

In all cases (see Table 1), the images resulting from the application of the proposed algorithm have averagely higher entropy, VIF, WPSNR, contrast, and homogeneity. This suggests that the proposed method improved information content, visibility, and human perceptual quality of the input image when compared to the existing methods. The tabulated results in Table 1, the extract of sampled zoomed areas in Figure 6, and comparison with classical HNN in Figure 7 also show results produced by the proposed method are superior. These shows that a modification of the HNN based on the geometric correlation of pixels improves the pixel gradient vector and ultimately optimizes the local energy function, which enhances the image information content while preserving image features. The standard deviation values in all cases, as presented in Table 1, show lower values compared to the corresponding benchmark algorithms. This indicates that the proposed method gives more consistent and predictable results than existing algorithms.

## 5. Conclusion

In this paper, we have presented a solution to the HNN convergence problem. The problem was solved by sequencing the activation of a continuous modified HNN based on the geometric correlation of features within various image hyperplanes via pixel gradient vectors and regulated geometric pixel gradient vectors. Solution to the problems was attained by regularizing proposed MHNNs under cohomology, which enables them to act as an unconventional filter for pixel spectral sequences. These enables shifting of the focus to both local and global optimizations to help strengthen feature correlations within each image subspace. The results of the proposed algorithm tested via the selected image performance evaluation metrics showed that including the variance of the pixel gradient vector optimizes local and global minima of the energy function, which subsequently increases the perceived image quality. For future studies, we have anticipated an extension to video graphics, as well as hyperspectral and natural images, our research will consider combining these techniques with other algorithms.

## Figures and Tables

**Table 1:** Comparison of mean *μ* and standard deviation *σ* of performance evaluation metrics of the proposed method and existing state-of-the-art algorithm.

Algorithm		Entropy	VIF	WPSNR	Contrast	Homogeneity
MOBA [60]	*μ*	6.9633	0.6973	18.757	0.8738	0.8524
	*σ*	±0.4113	±0.5603	±5.7184	±0.7250	±0.0514
Proposed	*μ*	7.5011	2.6666	18.8048	1.7137	0.8905
	*σ*	±0.34639	±1.9890	±3.1614	±0.2770	±0.0292
ABC [59]	*μ*	7.3544	0.8019	23.835	0.7327	0.8770
	*σ*	±0.4400	±0.1730	±4.2695	±0.1824	±0.0003
Proposed	*μ*	7.7311	1.2826	24.4	1.8141	0.8987
	*σ*	±0.4448	±0.1375	±4.0574	±0.5766	±0.0116
WCO [58]	*μ*	6.6093	3.9243	20.6034	0.6098	0.8525
	*σ*	±0.8891	±2.6601	±4.5830	±0.2183	±0.0525
PSO [61]	*μ*	6.2710	2.3258	18.7493	0.3043	0.9013
	*σ*	±0.7374	±0.1.2584	±9.6409	±0.1738	±0.0373
Proposed	*μ*	7.2647	4.5668	28.8092	1.2119	0.9436
	*σ*	±0.4600	±2.7800	±1.6148	±0.4897	±0.02765

## Data Availability

The data used in this study is available in the references of the manuscript.
